# Exome sequencing and genome-wide association analyses unveils the genetic predisposition in hydroxychloroquine retinopathy

**DOI:** 10.1038/s41433-024-03044-x

**Published:** 2024-03-28

**Authors:** Hsun-I Chiu, Hui-Chen Cheng, Chih-Chiau Wu, Shih-Jen Chen, De-Kuang Hwang, Yi-Ming Huang, Yu-Bai Chou, Po-Kang Lin, Tai-Chi Lin, Ko-Hua Chen, Pei-Yu Lin, Yu-Fan Chang, An-Guor Wang

**Affiliations:** 1 Department of Ophthalmology, Taipei Veterans General Hospital, Taipei, Taiwan; 2 Department of Ophthalmology, School of Medicine, National Yang Ming Chiao Tung University, Taipei, Taiwan; 3 Program in Molecular Medicine, College of Life Sciences, National Yang Ming Chiao Tung University, Taipei, Taiwan; 4 Department of Life Sciences and Institute of Genome Sciences, College of Life Sciences, National Yang Ming Chiao Tung University, Taipei, Taiwan; 5 Brain Research Center, National Yang Ming Chiao Tung University, Taipei, Taiwan

**Keywords:** Genetics, Eye diseases

## Abstract

**Objectives:**

To unveil the candidate susceptibility genes in chloroquine/hydroxychloroquine (CQ/HCQ) retinopathy using whole exome sequencing (WES) and genome-wide association study (GWAS).

**Methods:**

Patients with a diagnosis of CQ/HCQ retinopathy based on the comprehensive demographic and ocular examination were included. The peripheral blood was extracted for WES and GWAS analyses. The Chinese Han Southern database from 1000 genomes was used as control group to compare the affected percentage. Multivariate logistic regression analysis adjusted for age, HCQ dose, duration and renal disease were used to analyze the correlation between genetic variants and visual outcome. A poor vision outcome was defined as visual acuity <6/12. An abnormal anatomical outcome was defined as disruption of ellipsoid zone in the fovea.

**Results:**

Twenty-nine patients with an average age of 60.9 ± 13.4 years, treatment duration of 12.1 ± 6.2 years, daily dose of 8.5 ± 4.1 mg/kg, and the cumulative dose of 1637.5 ± 772.5 g, were genotyped. Several candidate genes associated with CQ/HCQ retinopathy were found, including RP1L1, RPGR and RPE65, with a difference of affected percentage over 50% in mutation between the case and control groups. New foci in CCDC66: rs56616026 (OR = 63.43, p = 1.63 × 10^−8^) and rs56616023 (OR = 104.7, *p* = 5.02 × 10^-10^) were identified significantly associated with HCQ retinopathy. Multivariate analysis revealed increased genetic variants were significantly associated with poor functional (OR = 1.600, *p* = 0.004) and structural outcome (OR = 1.318, *p* = 0.043).

**Conclusions:**

Several candidate susceptibility genes including RP1L1, RPGR, RPE65 and CCDC66 were identified to be associated with CQ/HCQ retinopathy. In addition to disease susceptibility, patients with increased genetic variants are more vulnerable to poor visual outcomes.

## Introduction

Chloroquine or hydroxychloroquine (CQ/HCQ) is widely used in the treatment of autoimmune disease since 1950s, due to well-tolerated side effects, and ability to reduce the disease activity. [1] However, it may cause ocular toxicity and lead to irreversible vision loss. CQ retinopathy was first described in 1959 [2], and HCQ retinopathy was first reported in 1967 [3]. Early toxicity is often asymptomatic. Once symptomatic, patients complain of reduced vision, glare, flash lights and constricted visual field. Abnormalities of the retinal pigment epithelium (RPE) are clinically detected in the paramacular area, often sparing the fovea. Asians may have extended perimacular pattern of RPE abnormalities [4]. The “flying saucer” sign is seen on spectral-domain optical coherence tomography (SD-OCT), which describes paramacular/perimacular ellipsoid zone (EZ) loss with a preservation of EZ in the fovea. Fundus autofluorescence (FAF) reveals hyper-autofluorescence as early RPE damage, and hypo-autofluorescence in the atrophic RPE area. Visual field (VF) typically detects a cecocentral/paracentral scotoma. Multifocal electroretinography (mfERG) may demonstrate depression in the paramacular or perimacular area. Bull’s eye maculopathy develops in more advanced toxicity, with concentric parafoveal RPE loss. As RPE atrophy gradually involves the fovea and entire retina, it leads to severe loss of vision. Although the ocular toxicities of CQ and HCQ are well established and easily detected by modern technologies, the associating symptoms are rather obscure to be noticed by patients at an early stage.

The prevalence risk of CQ/HCQ retinopathy has been estimated to be 0.65% in the first year and increased to 3.1% after 20 years of use [1, 5]. With the progress of using new modalities, such as SD-OCT, automated VF perimetry, FAF, and mfERG, the overall prevalence of retinopathy has been found to be 7.5% in patients who have used the medication for over 10 years, and prolonged use would increase the risk to 20% after 20 years use. [6, 7] The American Academy of Ophthalmology (2016 revision) recommends limiting daily HCQ use to less than 5.0 mg/kg real weight to reduce the risk of retinopathy (OR 5.67; 95% CI, 4.14–7.79 for >5.0 mg/kg) [7, 8]. Other factors include preexisting maculopathy, use of tamoxifen or concomitant renal disease [8]. However, some cases of retinal toxicity have been reported in patients with minimal exposure and without risk factors, suggesting that other predisposing factors may be involved [9]. The possibility of genetic or molecular factors that predispose to CQ/HCQ toxicity is currently unknown.

Noah et al. reported ABCA4, ATP-binding Cassette Transporter Retina-specific (ABCR, ABCA4), might be the causative candidate gene to develop retinal toxicity when exposed to CQ/HCQ. In their study, two of eight patients had missense mutations of ABCA4, including 3385C>T, 3602T>G, and 6320G>A. Interestingly, one subject with triple missense mutations developed CQ/HCQ retinopathy after 1.5 years exposure to CQ within recommended daily dose [10]. Kalev and Columbia University group [11] reported eight genetically-confirmed Stargardt disease patients developed the SD-OCT phenotypic features of CQ/HCQ retinopathy without exposure to CQ/HCQ. It still remains unknown whether individual susceptibility to the CQ/HCQ retinal toxicity will be determined or affected by genetic variants. Thus, we proposed this study using exome sequencing and genome-wide association analyses (GWAS) to unveil novel candidate susceptibility genes in CQ/HCQ retinopathy.

## Methods

### Patient enrollment

This study was conducted according to the Declaration of Helsinki and approved by the Institutional Review Board for Human Research of Taipei Veterans General Hospital (2020-08-006B and 2022-12-002AC). Informed consent was obtained from each patient for the genetic testing. We reviewed patients who had visited our ophthalmology department in Taipei Veterans General Hospital, a tertiary hospital in Taipei, Taiwan, from September 2020 to December 2021. All charts and images until December 2021 or last visit were carefully reviewed. We enrolled patients with a current or previous chloroquine or hydroxychloroquine (CQ/HCQ) treatment, with a diagnosis of CQ/HCQ retinopathy based on the clinical and imaging evidence and with age between 20 to 80 years old. Patients diagnosed to have other retinopathies (e.g. retinitis pigmentosa (RP), cone rod dystrophy and …etc.), with family history of RP, age less than 20 or older than 80 years old were excluded.

### Diagnosis of HCQ retinopathy

Demographic data, including age, gender, medical comorbidities, CQ/HCQ daily dose and cumulative dose, medication duration before the time of CQ/HCQ cessation, ideal and actual body weight (ABW), renal function and liver function were collected. All patients underwent comprehensive ophthalmic examinations in both eyes, including best-corrected visual acuity (BCVA, using Snellen charts), slit lamp, SD-OCT (Optovue Inc., Fremont, CA), fundus autofluorescence (FAF, excitation 486 nm; emission > 500 nm; Canon Inc.) and visual field with 24-2 or 30-2 automated perimetry (Humphrey Field Analyzer; Carl Zeiss Meditec, Dublin, CA). Clinical diagnosis was made based on CQ/HCQ exposure history, parafoveal/perifoveal/diffuse pattern of RPE changes on autofluorescence, loss of outer retinal layers in ellipsoid zone (EZ) or external limiting membrane (ELM) on SD-OCT, and cecocentral or central scotoma or generalized constriction on visual field. RPE changes in FAF were categorized as parafoveal, perifoveal, or diffuse involvement when the RPE disruption occurs in 2–6 degrees from the central of the foveal, in more than 6 degrees from the central of the foveal, or mixed with parafoveal and perifoveal area, respectively [12]. Multifocal ERG, recommended for the early detection of HCQ retinopathy, was not routinely performed in our study due to the higher prevalence of advanced cases of HCQ retinopathy. Clinical charts and images were carefully reviewed by two ophthalmologists, AG Wang and HI Chiu.

### Genotyping and exome sequencing

To identify the causative candidate gene associated with HCQ retinopathy, exome sequencing was performed according to the referenced paper as the following. [13] Genomic DNA was extracted from peripheral blood for genetic analysis. Exome sequencing was conducted on 1000 ng of genomic DNA from participants. Fragment libraries were prepared from the sheared samples by sonication and target enrichment will be performed according to the manufacturer’s protocols (SureSelect Human All Exon V6 kit). Captured DNA was amplified followed by solid‐phase bridge amplification and paired‐end sequenced on Illumina NovaSeq 6000 (Illumina, Inc.). Alignment of reads to the human reference sequence (hg38 assembly) and variants detection was performed using Genome Analysis Toolkit 4.1 (GATK, www.broad institute.org/gatk). The variant annotation information was obtained from Variant Effect Predictor (VEP) (https://www.ensembl.org/info/docs/tools/vep/index.html) and novel variants were filtered against 1000 Genomes (1000 genomes release phase 3, http://www.1000genomes.org/), dbSNP (http://www.ncbi.nlm.nih.gov/projects/SNP/snp_summary.cgi), and Genome Aggregation Database (gnomad.broadinstitute.org). A total of 917 comprehensive genes of interest from Online Mendelian Inheritance in Man® (OMIM) were included, which were associated with inherited retina diseases (IRD) including achromatopsia, congenital stationary night blindness, Leber congenital amaurosis, retinitis pigmentosa, cone rod dystrophy, cone dystrophy and macular dystrophy. PCR and Sanger sequencing will be finally applied to validate the mutation. Allele frequency <0.05 was detected to be significantly different from the downstream analysis. To eliminate the common variant in Han Chinese, the Chinese Han Southern database (CHS) from 1000 genomes was used as control groups. The affected percentage was determined by dividing the numbers of mutations by the total case numbers in the HCQ group or control group and standardized. The difference of affected percentage was calculated by subtracting the affected percentage of mutations in the controls from the affected percentage of mutations in the cases.

### Genome-wide association study (GWAS)

Variant calling was described as above and quality control was applied. The control group comprises 100 samples sourced from the 1000 Genomes Chinese Southern (CHS) population. We excluded those SNPs with heterozygosity outliner, with a total call rate of less than 95% in cases and controls combined, with a minor allele frequency (MAF) of less than 5%, with significant (*p* < 10^-5^) deviation from Hardy–Weinberg equilibrium in controls and SNPs in high linkage disequilibrium (LD) using pairwise method from PLINK with parameters of window size 50 kb, step size 5 and *R*^2^ threshold 0.8. Imputation was not performed due to concerns that it might homogenize the genomic profiles, making them more similar to the CHS population. PLINK (version 1.90b6.24) software was used for genetic analysis. Manhattan and quantile-quantile (Q-Q) plot was applied to represent the *P* values of the entire GWAS on a genomic scale. Principal components analysis (PCA) was performed to infer genetic variance between case and control groups. Genome-wide logistic regression was conducted for the SNPs. Differences were considered statistically at *p* value < 10^−4^ compared to the CHS group.

### Clinical outcome and statistical analysis

SPSS software (version 26.0.0) was used to perform all statistical analyses, including descriptive analysis, chi-square test, t test and logistic regression analyses. Outcome measures (BCVA and OCT) were examined at each patient’s latest visit. Poor vision was defined as a BCVA less than 6/12 (or 20/40) according to the American Academy’s Vision Rehabilitation Committee. [14] The cut off value of poor functional outcome was set at BCVA less than 6/12 in Snellen chart. SD-OCT images were obtained using horizontal and vertical lines through the fovea. The poor structural outcome was defined as disruption of ellipsoid zone in the 1500-μm-central foveal area on the OCT [15]. SPSS software (version 26.0.0) was used to perform all statistical analyses, including descriptive analysis, chi-square test, *t*-test and logistic regression analyses. A multivariate binary logistic regression, adjusted with age, HCQ dose, treatment duration and renal disease, was used to evaluate the correlation between genetic variants and poor functional/structural outcomes. Analysis of variance (ANOVA) was used to evaluate the association between variants and pattern of CQ/HCQ retinopathy. Factors with a *p* value of ≤0.05 in the analysis were considered statistically significant.

## Results

### Demographic factors and ocular characteristics

A total of 40 female and 1 male patients with HCQ retinopathy were enrolled in the present study. The demographic factors and ocular characteristics were listed in Tables 1 and S1, which was classified on patterns of RPE change: parafoveal, perifoveal, or mixed/diffuse pattern on autofluorescence. There was no statistical significance of age, HCQ dose and duration between 3 patterns. Patients with mixed or diffuse pattern of HCQ retinopathy had worse visual acuity and more macular disruption, compared to those with the perifoveal or parafoveal pattern. None of the patients received tamoxifen or had preexisting maculopathy. None had diabetic retinopathy. Three patients had chronic kidney disease, and none had hepatic disease. Twelve patients refused blood collection. The remaining 29 cases (28 female and one male patient) with age of 60.9 ± 13.4 (30–84) years, receiving HCQ treatment for 12.1 ± 6.2 (3–25) years, at daily dose of 8.5 ± 4.1 (3.0–19.1) mg/kg on actual body weight, with cumulative dose of 1637.5 ± 772.5 (292–3504) g, received genetic analysis. There were no statistically significant differences between age, daily dose on actual body weight, HCQ duration, cumulative dose, initial BCVA, final BCVA and ratio of foveal involvement between cases with (*n* = 29) or without gene analysis (*n* = 12) (Table S2).

**Table 1:** Clinical characteristics of patients with hydroxychloroquine retinopathy.

	Perifoveal (*n* = 6)	Parafoveal (*n* = 15)	Mixed or diffuse (*n* = 20)	*P* value
Age (years)	53.67 ± 15.65	63.64 ± 11.68	61.50 ± 14.14	0.326
Female/Male (F %)	6/0 (100%)	15/1 (93.8%)	20/0 (100%)	–
Daily HCQ dose (mg/kg of ABW)	6.21 ± 2.80	8.58 ± 1.90	8.67 ± 4.42	0.340
Duration (years)	10.17 ± 6.37	11.40 ± 3.16	13.90 ± 6.50	0.238
Latest BCVA (mean ± SD, logMAR) [range, Snellen]	0.06 ± 0.08 [6/5–6/7.5]	0.33 ± 0.31 [6/6–6/30]	0.83 ± 0.13 [6/6.7-HM]	<0.001^a^ Peri-Para 0.459 Para-Mix 0.007 Peri- Mix 0.001
Foveal involvement in SD-OCT (%)	0%	26.7%	55.0%	0.001^a^ Peri-Para 0.194 Para-Mix 0.028 Peri- Mix 0.001

Foveal involvement in SD-OCT was defined as disruption of ellipsoid zone in the central 1500-μm foveal area.

All values of *P* < 0.05 were deemed significant.

*HCQ* hydroxychloroquine, *ABW* actual body weight, *BCVA* best-corrected visual acuity, *HM* hand motion, *LP* light perception, *Mixed* parafoveal and perifoveal involvement, *SD-OCT* spectral-domain optical coherence tomography.

^a^Post hoc analysis.

### Exome sequencing study

**Fig. 1:**
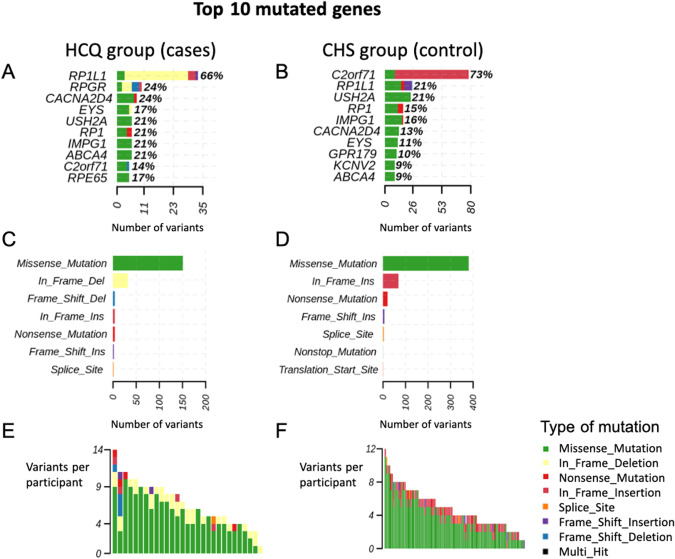
Inherited retinal disease associated genes with variants in the hydroxychloroquine retinopathy group (HCQ retinopathy group, as cases) and the Chinese Han Southern database (CHS group, as control). The top 10 mutated genes are shown for the cases (**A**) and control group (**B**). The percentage indicates the ratio of mutation in all samples. Counts of consequences of variants for the cases (**C**) and control group (**D**). Counts of variants per sample for the cases (**E**) and control group (**F**). The color of the bars indicates the consequences of the variants while each bar presents a sample. The colors green, red, orange, international orange, sky blue, and violet indicate the missense, nonsense, splice site, translation start site, frameshift deletion, and frameshift insertion, respectively. We found variants altered 100% (29 of 29 samples) in HCQ retinopathy group, with missense mutation as the major mutation type. HCQ group hydroxychloroquine retinopathy group, CHS group the Chinese Han Southern group, Del deletion, Ins insertion.

We genotyped 29 cases using 1000 ng of genomic DNA. After quality control, variant classification revealed 77.1% of missense mutation, 15.6% of in frame deletion, and 7.3% of frameshift deletion/in frame insertion/nonsense mutation/frameshift insertion/splicing. Single nucleotide polymorphism (SNP) was most frequent, with 79.5% compared to 2.9% of insertion and 17.6% of deletion. Among the causative candidate genes, the top 10 candidates were RP1L1 (66% of cases) followed by RPGR (24%), CACNA2D4 (24%), USH2A (21%), RP1 (21%), IMPG1 (21%), ABCA4 (21%), EYS (17%), RPE65 (17%) and C2orf71 (14%). We listed the frequency variants in Fig. 1. The Chinese Han Southern database (CHS) from 1000 genomes was performed as control groups to compare the affected percentage. RP1L1, RPGR, RPE65 had a difference more than 50% in mutation than controls; CACNA2D4, EYS, RP1, IMPG1 and ABCA4 had a difference of affected percentage less than 50%; USH2A and C2orf71 had a lower affected percentage in cases. The affected percentage differences were listed in Table 2.

**Table 2:** Candidate genes with high variant percentages in HCQ retinopathy.

Gene	Affected percentage (%)*		Difference (%)*
	Case (HCQ)	Control (CHS)	
RPGR	100.00	0.00	100.00
RPE65	100.00	0.00	100.00
RP1L1	75.73	24.2	51.53
ABCA4	69.69	30.31	39.38
CACNA2D4	65.00	35.00	30.00
EYS	61.05	38.95	22.10
RP1	57.97	42.03	15.94
IMPG1	56.39	43.61	12.78

*The affected percentage was determined by dividing the numbers of mutations by the total case numbers in the case group or control group and standardized. The difference of affected percentage was calculated by substracting the affected percentage of mutations in the controls from the affected percentage of mutations in the cases.

*HCQ* hydroxychloroquine retinopathy group, *CHS* the Chinese Han Southern group, *RPGR* Retinitis Pigmentosa GTPase Regulator, *RPE65* Retinoid Isomerohydrolase RPE65, *RP1L1* RP1-like Protein 1, *ABCA4* ATP-binding Cassette Transporter Retina-specific 4, *CACNA2D4* Calcium Channel Voltage-dependent Alpha-2/delta Subunit 4, *EYS* Eyes Shut Homolog, *RP1* RP1 Axonemal Microtubule-associated Protein, *IMPG1* Interphotoreceptor Matrix Proteoglycan 1.

### GWAS

**Fig. 2:**
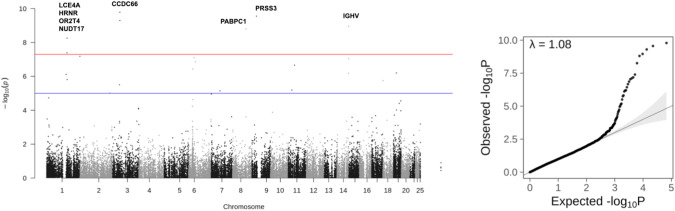
Manhattan plot of Genome-wide association study on 29 patients with hydroxychloroquine retinopathy and 100 population control participants from Chinese Han Southern (CHS) of the 1000 Genomes project. Principal components analysis (PCA) showed no outlier. The genomic inflation factor lambda (*λ*GC) was 1.08. The genome-wide significant signals are depicted in red (significant with cutoff value set at *P* < 5 × 10^−8^), whereas the suggestive variants are depicted in blue (significant with cutoff value set at *P* < 5× 10^−5^). The analysis identified SNPs associated with HCQ retinopathy, including Late Cornified Envelope Protein 4A (LCE4A), Hornerin (HRNR), Olfactory Receptor Family 2 Subfamily T Member 4 (OR2T4), Nucleoside Diphosphate-Linked Moiety X Motif 17 (NUDT17), Coiled-Coil Domain Containing 66 gene (CCDC66), Actin Related Protein 8 (ACTR8), Serine Protease 3 (PRSS3), Poly(A) Binding Protein Cytoplasmic 1 (PABPC1), Immunoglobulin Heavy Variable (IGHV).

To evaluate new risk foci associated with HCQ retinopathy, we genotyped 29 cases and used the Chinese Han southern database (CHS) of the 1000 genomes as control. SNPs with heterozygosity outlier, with a total call rate of less than 95% in cases and controls combined, with a minor allele frequency (MAF) of less than 5%, with significant (*p* < 10^−5^) deviation from Hardy–Weinberg equilibrium in controls were excluded. Compared to CHS, principal components analysis showed no outlier. The genomic inflation factor lambda (*λ*GC) was 1.08. Manhattan plot and Q-Q plot revealed traits with significant SNPs in Fig. 2. The analysis identified 12 SNPs associated with HCQ retinopathy, including Late Cornified Envelope Protein 4A (LCE4A, rs152709213), Hornerin (HRNR, rs152219743), Olfactory Receptor Family 2 Subfamily T Member 4 (OR2T4, rs248361751), Nucleoside Diphosphate-Linked Moiety X Motif 17 (NUDT17, rs145847528), Coiled-Coil Domain Containing 66 gene (CCDC66, rs56616026 and rs56616023), Actin Related Protein 8 (ACTR8, rs53876094), Serine Protease 3 (PRSS3, rs33796674), Poly(A) Binding Protein Cytoplasmic 1 (PABPC1, rs100706973), Immunoglobulin Heavy Variable 4–39 (IGHV4-39, rs106421729), IGHV3-38 (rs106410652) and IGHV1-2 (rs105986603). After the logistic regression analysis and functional annotation, two SNPs in Coiled-Coil Domain Containing 66 gene (CCDC66): rs56616026 (odds ratio, OR 63.43, *p* = 1.63 × 10^-8^) and rs56616023 (OR = 104.7, *p* = 5.02 × 10^−10^) were identified. CCDC66 accounted for 24% (7 patients) alteration in our HCQ cases, and the difference of affected percentage was 38% more in case groups. Among 7 patients, there were 1 perifoveal involvement and 6 mixed/diffuse patterns. The correlation between CCDC66 and FAF patterns was insignificant (*p* = 0.125). FAF images were presented in supplement Fig. 1.

### The clinical correlation between variants and visual outcome

The number of variants varied between patients, ranging from 2 to 15. The fundus photographs/FAF/OCT in patients with maximum and minimum number of variants were presented in supplementary fig. 2. Logistic regression analysis was performed to investigate the correlation between genetic variants and poor functional and structural outcome. After adjusting age, HCQ dose and duration, patients with more genetic variants (Odds ratio, OR = 1.410, *p* = 0.004) had higher risk of poor functional outcome (OR = 1.600, *p* = 0.004) and worse structural outcome (OR = 1.318, *p* = 0.043) **(**Table 3(A)). However, the association between number of genetic variants and three patterns of CQ/HCQ retinopathy was not revealed (*p* = 0.313) **(**Table 3B). The correlation between underlying genetic variants and age was insignificant (*p* = 0.650).

**Table 3:** The clinical correlation between variants and visual outcome.

(A) Multivariate logistic regression analysis of genetic variants associated with poor functional or structural outcome				
	Poor functional outcome^a^ (Odds ratio)	*p* value	Poor structural outcome^b^ (Odds ratio)	*p* value
Age	1.042	0.219	0.987	0.646
Dose on ABW	1.094	0.363	0.996	0.961
Duration	1.017	0.828	0.922	0.202
Renal disease	0.561	0.695	1.877	0.607
Genetic variants	1.600	0.004	1.318	0.043

(B) The correlation between number of genetic variants and patterns of hydroxychloroquine retinopathy (*n* = 58 eyes)		
	Variants (mean ± SD) [range]	*P* value
Perifoveal (*n* = 10)	5.8 ± 1.8 [4~9]	0.313*
Parafoveal (*n* = 16)	7.0 ± 2.0 [4~9]	
Mixed or diffuse (*n* = 32)	7.3 ± 3.2 [2~12]	

All values of *P* < 0.05 were deemed significant.

*BCVA* best corrected visual acuity, *ABW* actual body weight, *Mixed* parafoveal and perifoveal pattern.

*Post hoc analysis: No significance compared parafoveal pattern to perifoveal pattern (*p* = 0.520), parafoveal pattern to mixed pattern (*p* = 0.925), and perifoveal pattern to mixed pattern (*p* = 0.281).

^a^Poor functional outcome: the cutoff value was set at best-corrected visual acuity less than 6/12 in Snellen chart.

^b^Poor structural outcome was defined as disruption of ellipsoid zone in the central 1500 μm foveal area.

## Discussion

To the best of our knowledge, this report was the first study focused on the genetic susceptibility of HCQ retinopathy via exome sequencing and GWAS analysis. Despite satisfactory systemic safety of CQ/HCQ, evaluating predisposing factors prior to CQ/HCQ use may help to prevent retinal toxicity. In our study, 29 of 41 cases received peripheral blood extraction and genetic analysis. Several candidate genes associated with CQ/HCQ retinopathy were found with exome sequencing, including RP1L1, RPGR, RPE65, CACNA2D4, EYS, RP1, IMPG1 and ABCA4. Two novel SNPs in CCDC66 was identified by GWAS to be related to HCQ retinopathy. The results of the multivariate logistic regression analysis, adjusted for age, HCQ dose, duration and renal disease, revealed increased number of genetic variants were significantly associated with poor functional (OR = 1.600, *p* = 0.004) and structural outcome (OR = 1.318, *p* = 0.043). Genomic annotation of associated candidate susceptibility genes helps the understanding of genetic pathogenesis of CQ/HCQ retinopathy.

Using exome sequencing analysis, we found RP1L1, RPGR and RPE65 were the top 3 candidate susceptibility genes, with a difference of affected percentage over 50% in mutation between cases and controls. *RP1L1 (Retinitis Pigmentosa-1-like-1)* was identified in 19 patients (66%) with pathogenic mutations in our study. RP1L1 encodes two N-terminal doublecortin domains, which regulate microtubule polymerization. Its expression is related to photoreceptor development and mutations in this gene are associated with occult macular dystrophy and retinitis pigmentosa 88 [16, 17]. *RPGR (Retinitis Pigmentosa GTPase Regulator)* mutations were found in 24% (7 patients) of HCQ group and was absent in the control group. It is localized in the outer segments of rod and cone photoreceptors. RPGR mutations reduce the GDP/GTP exchange activity and disrupt cilia function and are associated with retinitis pigmentosa. [18, 19] *RPE65 (Retinoid Isomerohydrolase 65)* was identified in 5 patients (17%) and was absent in the control group. RPE65 converts all-trans retinyl ester to 11-cis retinol in the retinal pigment epithelium. RPE65 mutations have been identified in Leber congenital amaurosis and adult-onset retinitis pigmentosa. [20, 21] Nevertheless, these candidate genes need further validity for their effect in the pathogenesis of hydroxychloroquine retinopathy.

In the present study, we found *ABCA4 (ATP Binding Cassette Subfamily A Member 4)* mutations were present in 6 patients (21%) with 6 missense mutations in exon 1531G>A, 6498G>C, 5501A>G, 2123A>T, 1531G->A and 1433C>T. *ABCA4 (ATP Binding Cassette Subfamily A Member 4)* encodes an ATP-binding cassette (ABC) superfamily transmembrane protein in the retina photoreceptor cells, and mutations in this gene cause *Stargardt Disease 1, Retinitis Pigmentosa 19, Cone-Rod Dystrophy 3 and Age-related macular degeneration*. Previous study using direct sequencing of ABCA4 revealed two in eight HCQ patients had missense ABCA4 mutations, including c.3385C>T, c.6320G>A, and c.3602T>G [10]. They concluded that patients with ABCA4 mutation might be more susceptible to retinal toxicity when exposed to CQ/HCQ. However, inconsistent findings were reported in a study by Mack et al. [22], with only one variant SNP related to ABCA4 found in their study. To validate the deleterious effect of the ABCA4 variants, further evaluation is needed to assess their cellular effects and explore underlying mechanisms.

In GWAS analysis, we found 12 SNPs had a significant association (p < 10^-4^) with HCQ retinopathy in genes LCE4A, HRNR, OR2T4, NUDT17, CCDC66, PRSS3, PABPC1 and IGHV. After the logistic regression analysis and functional annotation, two SNPs in *Coiled-Coil Domain Containing 66 gene (CCDC66)*: rs56616026 (Odds ratio, OR 63.43, p = 1.63 × 10^-8^) and rs56616023 (OR = 104.7, *p* = 5.02 × 10^−10^) were identified. Exome sequencing revealed CCDC66 was present in 7 patients (24%, p7,9,12,25,31,35,37) with HCQ retinopathy and with a 38% higher difference of affected percentage. CCDC66 was first identified in humans, mice and dogs by Dekomien et al. [23] It encodes a microtubule binding protein for ciliogenesis and plays an important role in microtubule-dependent active transport, centriolar satellite distribution [24] and cooperative action of centriolar satellites, microtubules and molecular motors [25] in human retinal cells. The CCDC66 mutations in animal models have been associated with retinal degeneration [24, 26], with electrophysiologic finding of reduced scotopic a wave and photopic b wave. Its clinically associated disease remains unveiled in humans. Because animal models could not predict with sufficient certainty what will happen in humans, further study is needed to map CCDC66 in human ciliopathies, and to understand the prevalence, phenotype and pathogenesis of CCDC66-related disease.

In our study, the number of variants varied between patients, ranging from 2 to 15. We found the association between genetic variants and worse functional (OR = 1.600, *p* = 0.004) and structural outcome (OR = 1.318, *p* = 0.043). Thus, genetic variants may not only affect the individual susceptibility to the HCQ retinal toxicity, cumulated effects of variant load may also have an effect on the clinical outcome. Additionally, the extent of macular damage serves as a crucial factor in assessing the severity of visual acuity and the visual prognosis. Patients exhibiting diffuse atrophy typically experience a poorer visual prognosis compared to those with more localized or limited involvement. However, conventional fundoscopy is difficult to distinctly differentiate between the mixed type and diffuse pattern. It is noteworthy that ultra-widefield imaging, although not accessible in our hospital during the study period, holds the potential to offer enhanced pericentral and peripheral details [27]. Consequently, we amalgamated the mixed pattern and diffuse pattern categories for prognosis assessment. Further studies with a larger number of participants may provide more insight into the variant spectrum and its clinical correlation.

Our study had some limitations. First, there was a lack of a control group that matched the age and diagnosis of the patients receiving CQ/HCQ without CQ/HCQ retinopathy. The prevalence of the subjects who receiving CQ/HCQ in the CHS control group was unknown. The genetic predisposition to develop autoimmune disease, which is the cause for these patients to take CQ/HCQ, is not excluded by the current study. Nevertheless, since the inherited retina diseases (IRD)-related genes from OMIM were used as the targeting panel for exome sequencing analysis, the possibility of sorting out autoimmune disease-predisposing genes or drug metabolism genes is low. Secondly, genes that contribute to drug metabolism, such as cytochrome P450, are not included in current study, and further investigation is warranted to assess potential candidate genes related to drug metabolism. Third, the disparity in gender prevalence within autoimmune diseases might contribute to the observed gender imbalance in our cohort, because around 80% of individuals affected by autoimmune conditions are women [28, 29]. Factors, such as sex chromosome and hormone differences, warrant further exploration. Additionally, family history for retinitis pigmentosa could be unreliable and under-diagnosed, since RP is not a well-known eye disease and medical resources is limited. Last but not least, the correlations between the genotype and the early drug toxicity of the patients with CQ/HCQ retinopathy were not found in the study due to the higher prevalence of advanced cases of HCQ retinopathy. To detect early drug toxicity and associated underlying genetic defects, generalized screening of patients with HCQ/CQ use in larger sample size was recommended. Despite these limitations, this study was the first study that provided a better understanding on the genetic susceptibility of HCQ retinopathy via exome sequencing and GWAS analysis.

Supplemental material is available at Eye’s website.

## Summary

### What was known before

- Chloroquine or hydroxychloroquine (CQ/HCQ) is widely used in the treatment of autoimmune disease. Patients are vulnerable to toxic retinopathy with factors, including increased CQ/HCQ dosage, prolonged duration, preexisting maculopathy, use of tamoxifen or concomitant renal disease.

### What this study adds

- The possibility of genetic or molecular factors that predispose to CQ/HCQ toxic retinopathy is currently unknown. In this study, several candidate susceptibility genes including RP1L1, RPGR, RPE65 and CCDC66 were identified to be associated with CQ/HCQ retinopathy via exome sequencing and genome-wide association study. Patients with increased genetic variants are more vulnerable to poor visual outcomes. This study was the first study that provided a better understanding on the genetic susceptibility of CQ/HCQ retinopathy.

### Supplementary information

Supplement legends

Figure S1

Figure S2

Supplementary table

## Data Availability

The data presented in this study are available on request from the corresponding author, AG Wang. The data are not publicly available due to ethical restrictions.
