# Supplementary material for: Exome sequencing and genome-wide association analyses unveils the genetic predisposition in hydroxychloroquine retinopathy
Source: Eye (Lond). 2024 Mar 28;38(10):1926–32. doi: 10.1038/s41433-024-03044-x (PMC11226719; doi:10.1038/s41433-024-03044-x)
Supplement: Supplementary file 1 — Supplement legends [file 41433_2024_3044_MOESM1_ESM.docx]

Supplement

Supplement figure 1. Clinical presentations in fundus autofluorescence of *CCDC66*-associated hydroxychloroquine retinopathy.

Exome sequencing revealed CCDC66 mutations were present in 7 patients (24%), with 1 perifoveal involvement (p12) and 6 mixed/diffuse patterns (p7,9,25,31,35,37).

Supplement figure 2. Clinical presentations in fundus autofluorescence in patients with maximum number (P35, P7) and minimum number (P37, P31) of variants.
