# Supplementary figures and images for: Exome sequencing and genome-wide association analyses unveils the genetic predisposition in hydroxychloroquine retinopathy
Source: Eye (Lond). 2024 Mar 28;38(10):1926–32. doi: 10.1038/s41433-024-03044-x (PMC11226719; doi:10.1038/s41433-024-03044-x)

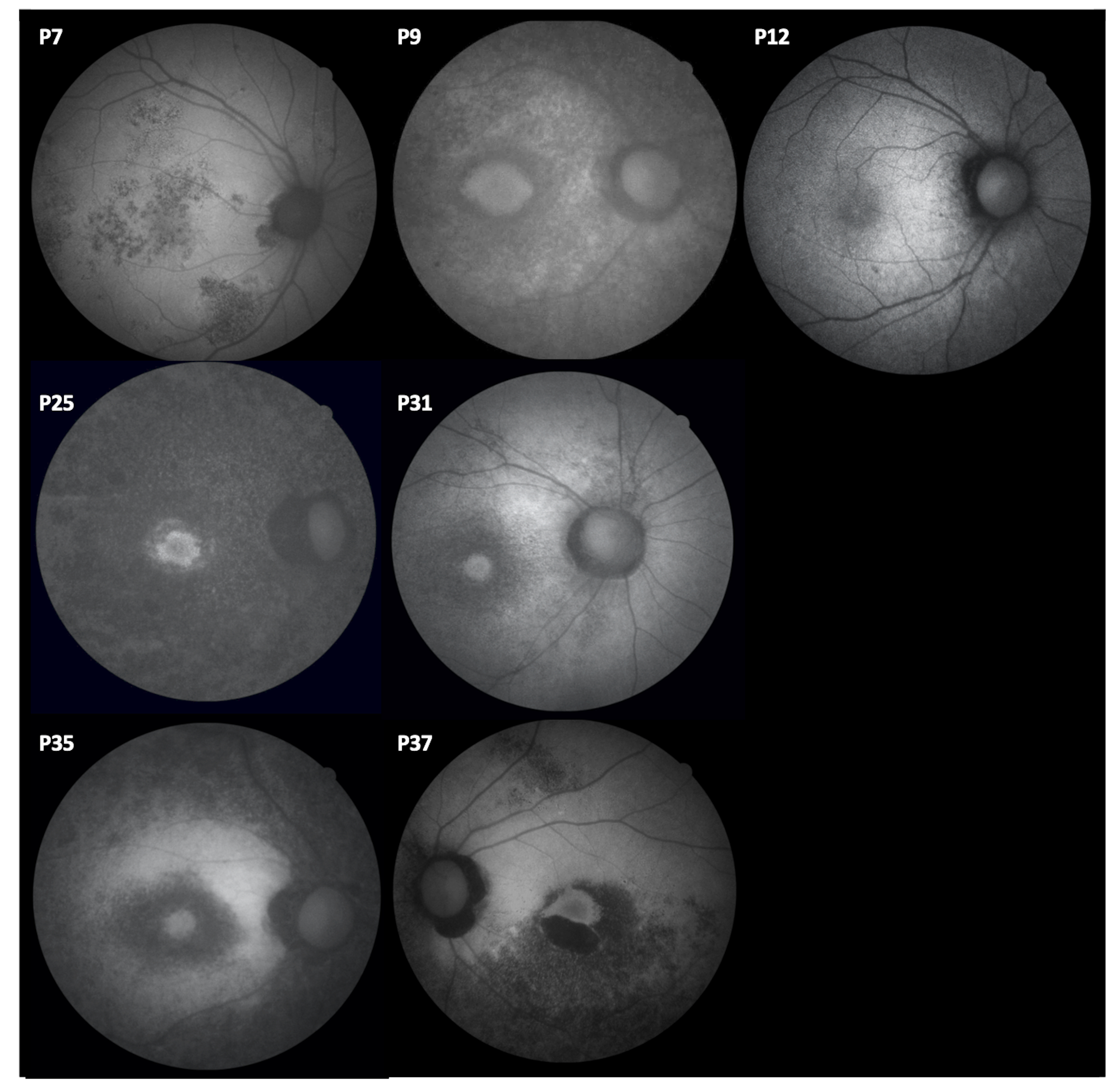

Supplement: Supplementary file 2 — Figure S1 [file 41433_2024_3044_MOESM2_ESM.jpg]

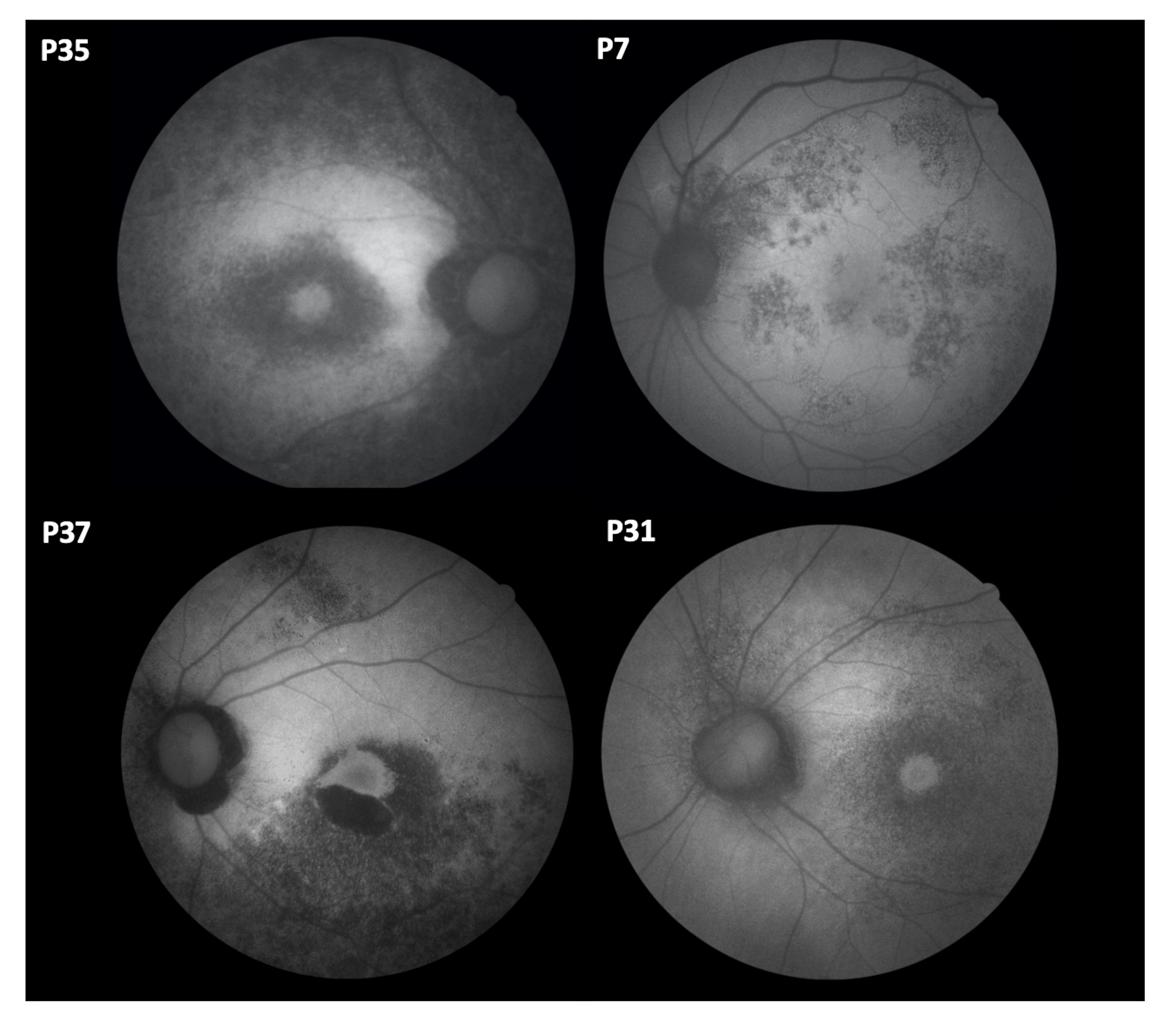

Supplement: Supplementary file 3 — Figure S2 [file 41433_2024_3044_MOESM3_ESM.jpg]
