# Supplementary material for: Exome sequencing and genome-wide association analyses unveils the genetic predisposition in hydroxychloroquine retinopathy
Source: Eye (Lond). 2024 Mar 28;38(10):1926–32. doi: 10.1038/s41433-024-03044-x (PMC11226719; doi:10.1038/s41433-024-03044-x)
Supplement: Supplementary file 4 — Supplementary table [file 41433_2024_3044_MOESM4_ESM.docx]

**Table S1.** Clinical characteristics of patients with hydroxychloroquine retinopathy

| **case** | age (years), gender (male or female) | Diagnosis | Daily dose of HCQ (mg/kg of actual body weight) | Duration  (years) | Snellen chart of visual acuity (OD/OS) | OCT of foveal involvement (OD/OS) | Pattern | Visual field |
| --- | --- | --- | --- | --- | --- | --- | --- | --- |
| 1 | 66 F | SjS | 9.76 | 13 | 0.8/0.8 | -/- | para | central |
| 2 | 62 F | RA | 7.10 | 15 | 0.9/0.9 | -/- | para+peri | central+paracentral |
| 3 | 57 F | SLE,SjS | 15.69 | 6 | 0.5/0.2 | +/+ | para+peri | diffuse |
| 4 | 69 F | RA | 8.00 | 24 | 0.5/LP | +/+ | diffuse | diffuse |
| 5 | 56 F | RA | 8.60 | 9 | 0.1/5/60 | +/+ | para | central |
| 6 | 30 F | dermotomyositis, scleroderma | 18.18 | 6 | 0.1/0.2 | +/+ | para+peri | central+paracentral |
| 7 | 65 F | SLE | 3.64 | 16 | 0.066/0.6 | +/- | para+peri, scattered | paracentral |
| 8 | 79 F | RA | 7.55 | 7 | CF/0.4 | -/- | para+peri | N/A |
| 9 | 70 F | RA | 4.56 | 9 | 0.8/0.8 | +/+ | para+peri | diffuse |
| 10 | 52 F | SjS | 8.33 | 15 | 0.8/0.8 | -/- | para | central |
| 11 | 62 F | RA | . | 11 | 0.1/0.1 | +/+ | para | central+peripheral rim |
| 12 | 40 F | SjS | 5.00 | 4 | 1.0/1.0 | -/- | peri | cecocentral |
| 13 | 48 F | SLE | 9.52 | 14 | 1.0/1.0 | -/- | para | central |
| 14 | 63 F | SLE | 8.89 | 5 | 0.6/0.7 | +/+ | para | central |
| 15 | 56 F | SLE | 4.26 | 13 | 1.0/1.2 | -/- | para | central |
| 16 | 64 F | SLE | 7.77 | 15 | 0.4/0.6 | -/- | para | central |
| 17 | 64 F | SLE | 8.70 | 9 | 0.0083/0.016 | +/+ | diffuse | diffuse |
| 18 | 57 F | SLE | 6.98 | 16 | 0.6/0.6 | -/- | para+peri | diffuse |
| 19 | 59 F | SLE | 10.00 | 9 | 0.8/0.6 | -/- | peri | constriction |
| 20 | 35 F | SLE | 8.89 | 11 | 1.0/1.0 | -/- | peri | partial arcuate |
| 21 | 76 F | SLE | 3.64 | 19 | 0.7/0.6 | +/+ | para+peri | central+paracentral |
| 22 | 84 F | RA | 8.89 | 13 | 0.3/0.2 | -/- | para | constriction |
| 23 | 81 F | RA | 7.69 | 20 | HM/1/60 | +/+ | para+peri | diffuse |
| 24 | 67 F | RA | 6.35 | 10 | 0.3/0.5 | -/- | para+peri | constriction |
| 25 | 40 F | SLE | 7.27 | 5 | 0.3/0.3 | +/+ | diffuse | constriction |
| 26 | 81 F | RA | . | 14 | 0.5/0.4 | -/- | para | central |
| 27 | 32 F | SLE | 8.00 | 17 | 0.6/0.7 | -/- | diffuse | constriction |
| 28 | 59 F | SLE | 7.55 | 17 | HM/HM | +/+ | diffuse | diffuse |
| 29 | 72 F | RA | 3.85 | 14 | 0.9/0.9 | -/- | peri | peripheral rim |
| 30 | 47 F | SLE | . | 14 | . | -/- | para | central |
| 31 | 55 F | RA | . | 23 | 0.4/0.4 | +/- | para+peri | central+paracentral |
| 32 | 55 F | SLE/DLE | 19.05 | 7 | 1.0/1.0 | -/- | para+peri | central+paracentral |
| 33 | 70 F | sarcoidosis | 9.09 | 9 | 0.033/HM | +/+ | diffuse | diffuse |
| 34 | 46 F | RA | 2.99 | 20 | 0.6/0.8 | -/- | peri | paracentral |
| 35 | 68 F | SjS | 4.88 | 25 | 0.05/CF | +/+ | para+peri | diffuse |
| 36 | 69 F | SjS | . | 10 | 0.8/0.6 | -/- | para | central |
| 37 | 74 F | RA | 10.81 | 18 | 0.4/0.4 | -/- | para+peri | constriction |
| 38 | 78 F | RA | 11.76 | 9 | 0.2/0.3 | -/- | para | N/A |
| 39 | 58 M | AS | 8.00 | 6 | 0.4/0.3 | -/- | para | partial arcuate |
| 40 | 80 F | RA | . | 10 | 1.0/0.9 | -/- | para | central+peripheral rim |
| 41 | 70 F | RA | 5.00 | 4 | 1.0/1.2 | -/- | peri | partial arcuate |
| SjS: Sjogren syndrome, RA: rheumatoid arthritis, SLE: systemic lupus erythematosus, AS: ankylosing arthritis  CF: counting finger, HM: hand motion, LP: light perception, para: parafoveal involvement, peri: perifoveal involvement  OCT of foveal involvement (+) was defined as disruption of ellipsoid zone in the central 1500 um foveal area, and vice versa.  Visual field was performed with 24-2 or 30-2 automated perimetry. | | | | | | | |  |

**Table S2**. Demographic and ocular characteristics between HCQ cases with or without gene analysis

|  | with gene analysis (n=29) | no gene analysis (n=11) | p value (95% CI) |
| --- | --- | --- | --- |
| Age at drug cessation (y/o) | 60.9 ± 13.4 | 62.9 ± 15.0 | 0.714 (-13.5~9.46) |
| Dose on ABW (mg/kg/day) | 8.50 ± 4.05 | 7.42 ± 2.08 | 0.313 (-0.88~3.15) |
| Cumulative dose (g) | 1637.47 ± 772.54 | 1752.00 ± 588.54 | 0.620 (-585~356) |
| HCQ duration (year) | 12.1 ± 6.16 | 13.2 ± 3.86 | 0.524 (-4.28~2.22) |
| BCVA (mean ± SD, LogMAR) | initial 0.31 ± 0.30  final 0.55 ± 0.64  rate 0.055 ± 0.14 | initial 0.28 ±0.43  final 0.57 ± 0.86  rate 0.096 ± 0.23 | rate 0.818 (-0.18~-0.22) |
| SD-OCT foveal involvement | initial 35.7%  final 39.3% | initial 26.1%  final 33.3% | 0.803 (-0.31~0.40)  0.800 (-0.41~0.32) |
| HCQ: hydroxychloroquine, ABW: actual body weight, BCVA: best corrected visual acuity,  SD-OCT: spectral-domain optical coherence tomography.  All values of P <0.05 were deemed significant. | | | |
